# Supplementary material for: How to Predict Outcomes from a Biofeedback and Pelvic Floor Muscle Electric Stimulation Program in Patients with Urinary Incontinence after Radical Prostatectomy
Source: J Clin Med. 2021 Dec 27;11(1):127. doi: 10.3390/jcm11010127 (PMC8745214; doi:10.3390/jcm11010127)
Supplement: Supplementary file 1 [file jcm-11-00127-s001.zip › jcm-1491896-supplementary.pdf]

Supplementary Table S1. Correlation coefficients (Spearman) among PAD test results and clinical or pathological characteristics of the population.

**Correlation**	**coefficient**	**P value**
Age - baseline PAD test	0.145148611	0.3674
Age - 12-week PAD test	-0.020745267	0.4755
Weight - baseline PAD test	0.071589066	0.2474
Weight - 12-week PAD test	-0.049614959	0.2758
Metabolic Syndrome - baseline PAD test	0.263940168	**0.0466**
Metabolic Syndrome - 12-week PAD test	0.192029357	0.0952
Metabolic Syndrome - PAD free	-0.243332713	**0.0418**
BMI - baseline PAD test	0.008827369	0.4782
BMI - 12-week PAD test	-0.036560345	0.4267
Prostate volume - baseline PAD test	0.421533652	**0.0269**
Prostate volume - 12-week PAD test	-0.476345434	**0.0213**
Prostate volume - PAD free	-0.301031099	**0.0429**
Pre-operative PSA - baseline PAD test	0.102564087	0.3756
Pre-operative PSA - 12-week PAD test	0.213037837	0.0847
NS procedure - baseline PAD test	-0.167332103	0.2186
NS procedure - 12-week PAD test	-0.184649498	0.2644
eLND - baseline PAD test	0.13908325	0.1753
eLND - 12-week PAD test	0.254885724	0.0682
ISUP grading - baseline PAD test	0.080339211	0.4218
ISUP grading - 12-week PAD test	0.151897219	0.3857
Staging pT - baseline PAD test	0.125334604	0.3842
Staging pT - 12-week PAD test	0.179430718	0.3724
Surgical technique - baseline PAD test	-0.091167213	0.4217
Surgical technique - 12-week PAD test	0.109611064	0.4372
Endovesical lobe - baseline PAD test	0.107310694	0.2476
Endovesical lobe - 12-week PAD test	0.152039635	0.2743

BMI = body mass index; PSA = prostate-specific antigen; eLND = extended lymph node dissection; ISUP = International Society of Urological Pathology; NS = nerve-sparing.

Supplementary Table S2. Comparison of characteristics of the population submitted to pelvic floor rehabilitation for urinary incontinence after radical prostatectomy on the basis of PAD free results at 3 -month interval [Mean±SD, median. Number of cases (%). *Two-tailed t test*].

	PAD status		p-value
	**PAD free**	**No PAD free**	
**Patients**, *n° (%)*	28 (38.9)	44 (61.1)	-
**Age** *(years)*			
mean± SD	64.8±4.9	66.3±4.9	0.3718
median	65	67	
**Weight** *(Kg)*			
mean± SD	82.3±7.7	80.9±10.0	0.5140
median	83	82	
**BMI**			
mean ± SD	25.9±2.2	25.4±2.3	0.6431
median	26	25	
**Metabolic Syndrome**, *n° (%)*			
- no	8 (28.6)	3 (6.8)	0.1051
- mild	17 (60.7)	34 (77.3)	
- full	3 (10.7)	7 (15.9)	
**Prostate Volume** *(cc)*			
mean± SD	39.7±15.8	50.7±19.2	0.0043
median	37.5	48.5	
**Presence of intravesical prostatic lobe**, *n° (%)*	5 (17.9)	11 (25.0)	0.4278
**Pre-operative total PSA** *(ng/ml)*			
mean± SD	8.1±3.2	7.9±4.2	0.9944
median	7.5	7.2	
**Post-operative total PSA** *(ng/ml)*			
mean ± SD	0.03±0.01	0.07±0.17	0.2481
median	0.03	0.03	
**Surgical technique at RP**, *n° (%)*			
- LRP	25 (89.3)	39 (88.6)	0.7658
- RARP	3 (10.7)	5 (11.4)	
**NS technique at RP**, *n° (%)*	9 (32.1)	6 (13.6)	0.1827
**eLND performed at RP**, *n° (%)*	4 (14.3)	10 (22.7)	0.3311
**Pathological stage (T)**, *n° (%)*			
- pT2	23 (82.1)	30 (68.2)	0.1732
- pT3a	4 (14.3)	11 (25.0)	
- pT3b	1 (3.6)	3 (6.8)	
**Positive SM at surgery (R1)**, *n° (%)*	2 (7.1)	7 (15.9)	0.4068
**ISUP grading**, *n° (%)*			
- 1	9 (32.1)	10 (22.7)	
- 2	12 (42.9)	19 (43.2)	
- 3	5 (17.9)	9 (20.4)	0.2919
- 4	2 (7.1)	5 (11.4)	
- 5	0	1 (2.3)	
**Rehabilitation: number of procedures**			
mean± SD	9.8±4.1	14.1±4.7	<0.0001
median	9.5	15	
**Rehabilitation: time length** *(weeks)*			
mean± SD	5.0±2.0	7.2±2.3	<0.0001
median	5	8	

**PAD weight at baseline** *(g)*			
mean ± SD	118.4±201.5	504.4±430.0	<0.0001
median	21.5	425.5	
**PAD weight at 2 weeks** *(g)*			
mean ± SD	38.7±54.6	286.6±276.6	<0.0001
median	10	200	
**PAD weight at 4 weeks** *(g)*			
mean ± SD	26.7±33.2	192.3±200.4	<0.0001
median	12	105	
**PAD weight at 6 weeks** *(g)*			
mean ± SD	8.2±16.7	125.3±123.3	0.0083
median	0	76	
**PAD weight at 12 weeks** *(g)*			
mean ± SD	0.2±0.8	75.3±97.9	<0.0001
median	0	27.5	
**PAD weight percentage reduction from baseline to 12 weeks**, %	99.8%	85.1%	

RP = radical prostatectomy; BMI = body mass index; PSA = prostate-specific antigen; LRP = laparoscopic RP; RARP = robot-assisted RP; eLND = extended lymph node dissection; ISUP = International Society of Urological Pathology; SM = surgical margins; NS = nerve-sparing.

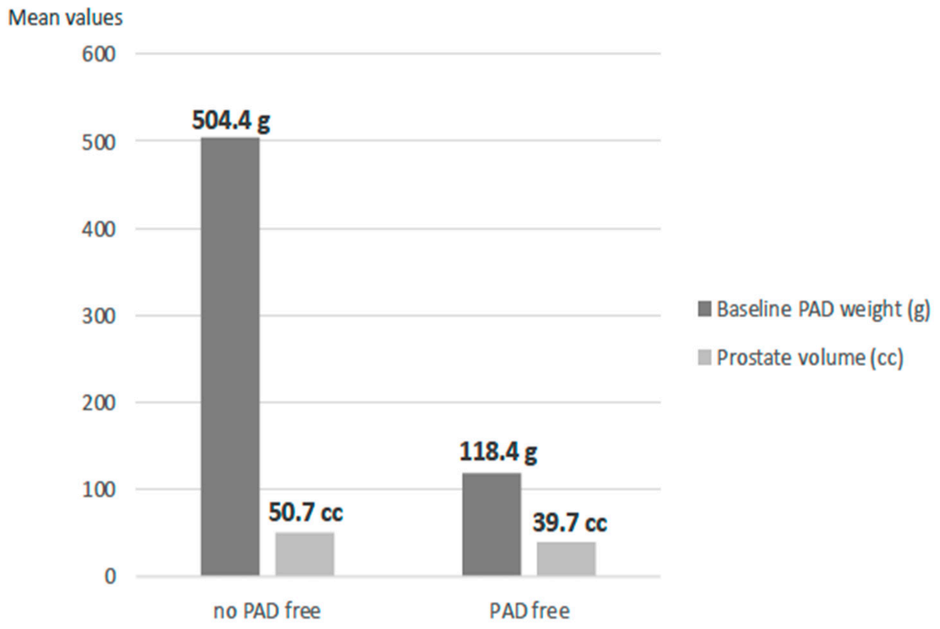

Supplementary Figure S1. Prostate volume (PV) (cc) and baseline pad weight (g) mean values according to final pad-free status. 338 × 190mm (150 × 150 DPI).
